# Comparison of Transcatheter Arterial Chemoembolization-Radiofrequency Ablation and Transcatheter Arterial Chemoembolization Alone for Advanced Hepatocellular Carcinoma with Macrovascular Invasion Using Propensity Score Analysis: A Retrospective Cohort Study

**DOI:** 10.1155/2020/1341863

**Published:** 2020-08-20

**Authors:** Yao Liu, Yuxin Li, Fangyuan Gao, Qun Zhang, Xue Yang, Bingbing Zhu, Shuaishuai Niu, Yunyi Huang, Ying Hu, Wei Li, Xianbo Wang

**Affiliations:** ^1^Center of Integrative Medicine, Beijing Ditan Hospital, Capital Medical University, Beijing 100015, China; ^2^Department of Gastroenterology, Dongzhimen Hospital, Beijing University of Chinese Medicine, Beijing 101121, China; ^3^Cancer Center, Beijing Ditan Hospital, Capital Medical University, Beijing 100015, China

## Abstract

**Background:**

To compare the efficacies of transcatheter arterial chemoembolization (TACE) with radiofrequency ablation (RFA) (TACE + RFA) and TACE alone in patients with hepatocellular carcinoma (HCC) and macrovascular invasion (MVI).

**Methods:**

In total, 664 patients having HCC with MVI were included. Of these patients, 141 were treated with TACE + RFA, 254 with TACE alone, and 269 with supportive therapy (control group). The overall survival (OS) was compared among these groups. Propensity score matching (PSM) was performed for balancing the characteristics of the three groups.

**Results:**

After one-to-one PSM, the 12-month OS rates were higher in the TACE and TACE + RFA groups than in the control group (*p*=0.0009 and *p*=0.0017, respectively). Furthermore, higher 12-month OS rates were observed in the TACE + RFA group than in the TACE group (*p*=0.0192). The 12-month OS rates of patients were remarkably higher in *α*-fetoprotein (AFP) < 400 ng/ml, tumor < 3, tumor diameter < 5 cm, or portal vein tumor thrombosis (PVTT) group who were treated with TACE + RFA than in those who were treated with TACE (*p*=0.0122, *p*=0.0090, *p*=0112, and *p*=0.0071, respectively).

**Conclusions:**

TACE + RFA provides a superior survival outcome than TACE alone in HCC patients, especially in AFP <400 ng/ml, tumor <3, tumor diameter <5 cm, or PVTT group.

## 1. Introduction

Globally, liver cancer is a major cause of cancer-related death, and hepatocellular carcinoma (HCC) accounts for >90% of all primary liver cancers [1]. Approximately, 10%–40% of HCC patients present with macrovascular invasion (MVI) of the portal and/or hepatic veins at the time of diagnosis [2–4]. MVI is an independent predictor of poor outcomes in patients with HCC. The median survival is considerably lower in HCC patients with MVI (2−4 months) than in those without MVI (10−24 months) [3]. Based on the Barcelona Clinic Liver Cancer (BCLC) staging system, HCC accompanied by MVI is defined as advanced HCC (BCLC stage C) [5–7]. Neither transcatheter arterial chemoembolization (TACE) with radiofrequency ablation (RFA) (TACE + RFA) nor TACE alone is suitable for treating these patients. Instead, the BCLC guidelines recommend sorafenib as a unique treatment for these patients. According to two the registered trials using sorafenib, the median overall survival (OS) changed from 5.6 to 8.1 months [8, 9]. In contrast, two randomized controlled trials reported that HCC patients with MVI did not respond well to sorafenib (response rate: 2%–3.3%).

Patients with BCLC stage C disease show high heterogeneity, and, therefore, the BCLC treatment algorithm cannot be applied regularly [6]. Kodama et al. retrospectively compared the effects of hepatic arterial infusion chemotherapy plus radiotherapy with those of sorafenib monotherapy in patients with portal vein tumor thrombosis (PVTT) and advanced HCC. The patients in the hepatic arterial infusion chemotherapy plus radiotherapy group showed remarkably longer median OS rates than those in the sorafenib monotherapy group (*p*=0.002) [10]. Several retrospective studies have reported that locoregional therapies, such as transarterial radioembolization and TACE, can affect PVTT progression and intravascular tumors developments [11–13]. However, the optimal treatment for HCC patients with MVI is unknown.

Currently, the number of studies that directly compare the treatment effects of TACE + RFA with TACE in HCC patients with MVI is insufficient. Therefore, in this study, a retrospective study was performed to compare the effects of the two aforementioned therapies on advanced HCC with MVI. Furthermore, propensity score matching (PSM) was performed to correct the potentially confounding elements that influence the effects of these therapies. PSM was also used to reach an equilibrium on the baseline characteristics of the two aforementioned groups [14].

## 2. Materials and Methods

### 2.1. Patients Selection

HCC was diagnosed according to the American Association for the Study of Liver Diseases criteria [5]. CT or MRI was used for assessing the presence of MVI. These techniques were used because intraluminal masses that expand into the portal vein, hepatic vein, and/or inferior vena cava show enhancement in the arterial phase, and, under low-attenuation, the intraluminal masses are enhanced in the portal phase [15, 16]. The requirement for informed consent from the patients was waived because of the retrospective nature of the study.

**Figure 1:**
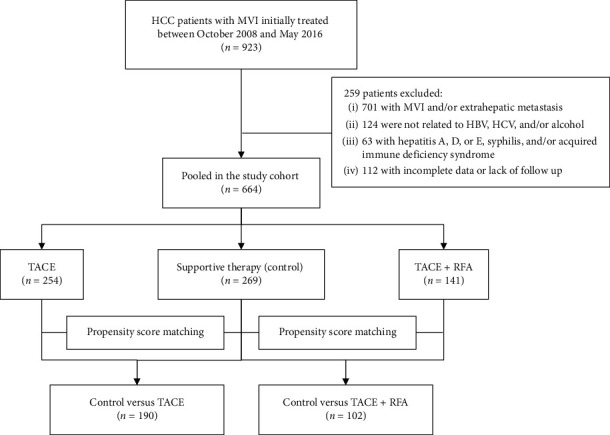
Flowchart of the treatments included in the study.

In total, 923 HCC patients with MVI were treated with TACE + RFA, TACE, or conservative therapy from October 2008 to May 2016. Among these patients, those who met the following criteria were included in this study: (i) receiving TACE + RFA, TACE, or conservative treatment as first-line therapy, (ii) without current or prior malignancies other than HCC, and (iii) availability for follow-up after the intervention. Finally, we recruited 664 patients who received TACE + RFA (*n* = 141), TACE (*n* = 254), or conservative treatment (*n* = 269) (Figure 1).

### 2.2. Characteristics of the Study Participants

The baseline demographic characteristics of the patients after TACE, TACE + RFA, or conservative treatment were compared, and the results are shown in Table 1. No significant difference in age distribution was seen among the three groups (*p*=0.657). Majority of the patients were male and positive for hepatitis B virus surface antigen and had a family history of HCC. The *γ*-glutamyl transpeptidase (GGT) levels were significantly higher in the control group than in the TACE and TACE + RFA groups (*p*=0.001 for both). The *α*-fetoprotein (AFP) levels were significantly lower in the TACE + RFA group than in the control group (*p*=0.018). Patients who received conservative treatment had significantly higher Child-Pugh class, model for end-stage liver disease (MELD) score, a large number of tumors, and high BCLC stages (*p* < 0.001 for all).

### 2.3. Propensity Score Matching

For one-to-one comparison between patients in the TACE and conservative treatment groups, variables in the propensity score model included GGT levels, MELD score, Child-Pugh class, white blood cell counts, tumor number, and BCLC stage. For one-to-one comparison between the patients in the TACE + RFA and control groups, the propensity score model included GGT levels, Child-Pugh class, AFP levels, tumor number, and BCLC stage as variables (Table 1). After propensity score matching (PSM), the significantly related characteristics were well-balanced (Table 2). For one-to-one comparison between patients in the TACE + RFA and TACE groups, variables in the propensity score model included tumor number, Child-Pugh class, AFP, and PVTT Vp4. After propensity score matching (PSM), the significantly related characteristics of the 139 pairs were well-balanced (Table 3).

### 2.4. Treatment Strategy

The appropriate treatment was selected by our multidisciplinary team. For the unresectable HCC patients with MVI, TACE has been the preferred palliative treatment. The following criteria were used for TACE alone: Child-Pugh A or B liver function, absent massive ascites, or with gross classification of diffuse type. Indications for TACE-RFA were Child-Pugh A or B liver function, absent massive ascites, or severe hypersplenism and were performed in patients with inoperative solitary or multiple tumors with a diameter of 3–7 cm. The HCC patients with MVI in the control group were evaluated as unsuitable for TACE or TACE-RFA therapy, and the patients with Child-Pugh A liver function gave up sorafenib treatment on their own.

Patients with HCC and MVI in the control group gave up sorafenib treatment.

### 2.5. TACE Procedure

Superior mesenteric angiography and common hepatic angiography were performed before chemoembolization to assess tumor vascularity, vascular anatomy, and tumor range. After administering local anesthesia to the patients, the Seldinger technique was adopted to introduce a 5F catheter into the abdominal aorta through the superficial femoral artery. During hepatic arterial angiography, fluoroscopy was performed to introduce the catheter into the celiac and superior mesenteric arteries, followed by identification of the feeding artery and staining of the tumor and the surrounding vascular anatomy. A microcatheter was introduced into the feeding artery via the catheter. A combination of ultra-fluid lipiodol (5–10 ml), lobaplatin (20–40 mg), and pirarubicin (10–30 mg) was then introduced into the tumor. If there was a significant arterioportal (AP) shunt, it is necessary to embolize gelatin sponge particles for occluding the shunt. Additional angiography was performed before completing the operation to ensure full blockage of the supplying artery.

### 2.6. RFA Procedure

RFA was performed one week after TACE treatment session, and under conscious analgesic sedation by intravenous administration of 0.5 mg atropine, 0.1 g pethidine hydrochloride, and 10 mg diazepam and application of local anesthesia (5 mL of 1% lidocaine). CelonPOWER RFA system (Olympus, Beckman Coulter, Inc.) with unipolar ablation electrode was used for ablation. All RFA procedures were performed percutaneously under nonenhanced CT by two of the four ablation experts with 6–15 years of experience. Certain needle position was determined by the deposition of lipiodol after TACE and the preoperative contrast-enhanced CT (CECT) or enhanced MRI. The number of ablations per procedure and whether the ablations were performed synchronously or in an overlapping manner was depended on the diameter, location, and shape of tumors. The aim was to achieve an ablative margin of at least 0.5 cm in the normal tissues surrounding the tumor, with the exception of subcapsular and perivascular portions. Before completion of the procedure, the needle tract was ablated to avoid bleeding and tumor spread.

### 2.7. Adjunct Treatments

In the follow-up period, for patients who met the antiviral treatment standard, we applied tenofovir, entecavir, lamivudine, telbivudine, or adefovir based on the virus replication degree and economic position. Based on the patients' liver function, prothrombin activity, and plasma albumin level, we applied liver protection drugs, plasma, and human albumin support treatment. Considering the patients' condition, we applied diuretics and vitamins. Antibiotics were applied to patients with spontaneous peritonitis. It was necessary to actively treat some complications such as hepatorenal syndrome, hepatic encephalopathy, and upper gastrointestinal bleeding.

### 2.8. Data Collection

Some prognostic factors related to the estimation of patient survival were assessed, such as age, gender, etiology (antihepatitis C antibody, hepatitis B antigen, and alcohol consumption), total bilirubin, GGT levels, serum albumin levels, Child-Pugh class, model for end-stage liver disease scores, MVI type (PVTT, hepatic vein tumor thrombosis (HVTT)), AFP levels, number of tumors, maximum size of the tumor, and BCLC stage.

### 2.9. Follow-Up

The study mainly focused on the survival of the patients which was estimated (in months) from the date of initial intervention to death or final follow-up. Three to six weeks after the first TACE or TACE + RFA treatment session, CT or enhanced MRI was performed to evaluate effect of treatment and detect the residual viable tumor. It is necessary to carry out tumor markers, CT, MRI, or ultrasonography every 1–3 months from baseline to 12 months for detecting local recurrent lesions as well as new intrahepatic lesions in an early stage.

In the TACE group, if thick lipiodol deposition and necrosis were observed in the liver tumor, and there was no tumor enlargement or new lesions, subsequent TACE sessions can be postponed. Responding to prior treatment, liver function and changes in PS determined the frequency of following TACE.

In the TACE + RFA group, there are two possible types of responses. The first is the complete response, in which the CECT or enhanced MRI is not enhanced in the area where the tumor lies in the arterial phase. The second is the incomplete response, and CECT or enhanced MRI is enhanced in the arterial stage, suggesting residual tumors [17]. It is suggested to carry out repeated TACE + RFA treatment for residual tumor patients following the initial combination therapy. If a residual tumor can be observed following two combination therapy sessions, combination therapy has failed. Patients would be switched to other treatments such as TACE alone or conservative treatment according to the features of the recurrent tumor, liver function status, and individual patient requirements.

### 2.10. Statistical Analysis

The continuous variables are presented as the mean ± standard deviation (if normally distributed) or median and range (if nonnormally distributed), and the categorical variables are presented as the number and percentage. To reduce bias related to the fact that patients were not randomized to receive TACE + RFA, TACE, or conservative treatment, the study used logistic regression to generate propensity scores for all the patients. This was because the 3 treatment groups could have confounding differences at baseline. The patients in the 3 treatment groups were matched with those in the control group according to the generated PSs, with a caliper width of 0.15 [14]. Following matching, the baseline covariates were compared using a paired *t*-test or Mann–Whitney *U* test for continuous variables and chi-square test for categorical variables. The Kaplan–Meier method was used for analyzing the OS. All analyses were performed using the SPSS 22.0 statistical package (SPSS, Inc., Chicago, IL, USA) and RMS packages (R version 3.0.2). A *p* value <0.05 was considered statistically significant.

## 3. Results

**Figure 2:**
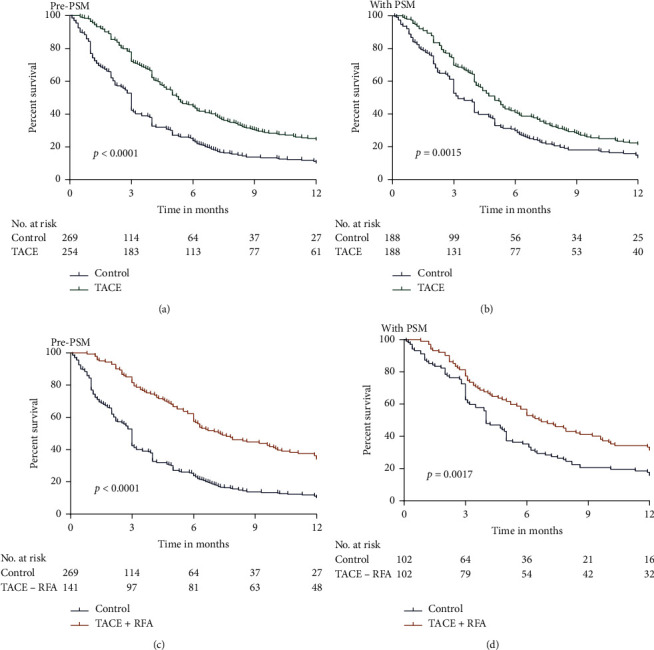
Comparison of the OS of patients who were treated with TACE and TACE + RFA with that of those who received conservative treatment before and after PSM analysis. (a) OS in TACE versus control group before PSM. (b) OS in TACE versus control group after PSM. (c) OS in TACE + RFA versus control group before PSM. (d) OS in TACE + RFA versus control group after PSM.

The median survival period for the patients after TACE was 5.3 months, TACE + RFA was 7.2 months, and control was 3 months before PSM and 5 months, 6.6 months, and 3 months, respectively, after PSM. The survival rates of TACE group were 72.0% at 3 months, 44.5% at 6 months, and 24.9% at 1 year (Figure 2(a)); the TACE + RFA group were 81.6% at 3 months, 57.4% at 6 months, and 34.0% at 1 year (Figure 2(c)); and the control group were 42.4% at 3 months, 23.8% at 6 months, and 10.0% at 1 year (Figure 2(a)).

### 3.1. Survival Analysis

**Figure 3:**
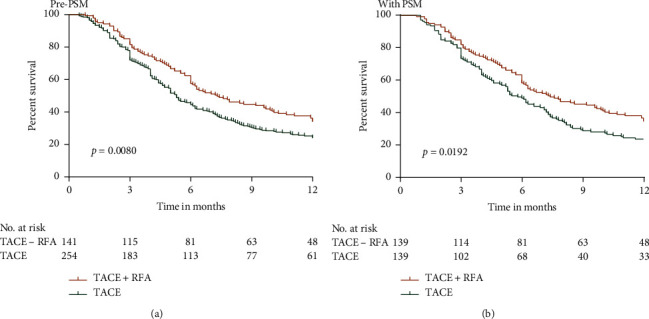
OS curves of patients in the TACE and TACE + RFA groups. (a) Before PSM and (b) after PSM.

Before PSM, Kaplan–Meier analysis showed that the TACE and TACE + RFA groups exhibited significantly higher OS than the control group (*p* < 0.0001 for all; Figures 2(a) and 2(c). One-to-one PSM helped in obtaining 188 pairs of patients in the control versus TACE groups, and significantly higher OS rates were observed in the TACE group than in the control group (*p*=0.0015, Figure 2(b)). In addition, 102 pairs of patients in control versus TACE + RFA groups (one-to-one matched) were formed, and significantly higher OS rates were observed in the TACE + RFA group than in the control group (*p*=0.0017,Figure 2(d)). Furthermore, the 12-month survival rates related to TACE + RFA and TACE treatments were analyzed. Our results showed that OS rates were significantly higher in the TACE + RFA group than in the TACE group before and after PSM (*p*=0.0080 and *p*=0.0192, respectively; Figure 3).

**Figure 4:**
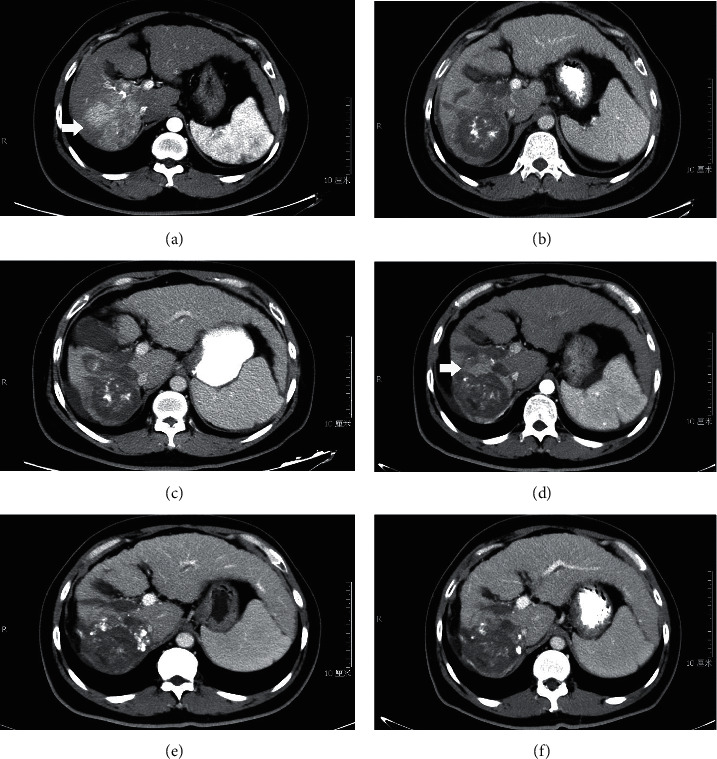
Images of diagnosis and follow-up of a 37-year-old patient with massive HCC and PVTT. (a) CT showing tumor and thromboses in the right branch of portal vein (arrow). (b, c) CT showing no tumor and PVTT enhancement at 1 month and 3 months after first TACE + RFA. (d) CT showing tumor enhancement at 5 months after first TACE + RFA (arrow). (e, f) CT showing no tumor enhancement at 2 months and 5 months after second TACE + RFA.

Figure 4 shows the CT images of a typical patient with massive HCC and PVTT before and after TACE + RFA treatments.

### 3.2. Subgroup Analysis

**Figure 5:**
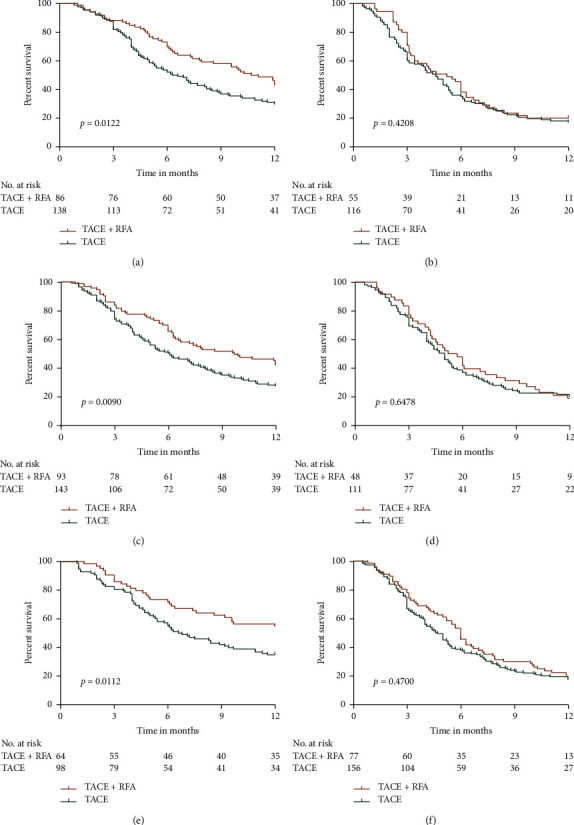
OS of patients in the TACE + RFA group compared with the TACE group in subgroups. (a) OS in AFP < 400 ng/ml group; (b) OS in AFP ≥ 400 ng/ml group; (c) OS in tumor number < 3 groups; (d) OS in tumor number ≥ 3 groups; (e) OS in tumor diameter < 5 cm group; and (f) OS in tumor diameter ≥ 5 cm group.

We further analyzed the 12-month OS in HCC patients with AFP <400 ng/ml or AFP ≥400 ng/ml, tumor number <3 or tumor number ≥3, and tumor diameter <5 cm or tumor diameter ≥5 cm who underwent TACE + RFA or TACE alone. Our results demonstrated that the 12-month OS after TACE + RFA was significantly higher than TACE alone in the AFP <400 ng/ml, tumor number <3, and tumor diameter <5 cm groups (Figures 5(a), 5(c), and 5(e), *p*=0.0122, *p*=0.0122, *p*=0.0090, and *p*=0.0112), and that the 12-month OS was similar in the AFP ≥ 400 ng/ml, tumor number ≥ 3, and tumor diameter ≥ 5 cm groups after TACE + RFA or TACE alone (Figures 5(b), 5(d), and 5(f), 0.4208, *p*=0.6478, and *p*=0.4700, respectively).

**Figure 6:**
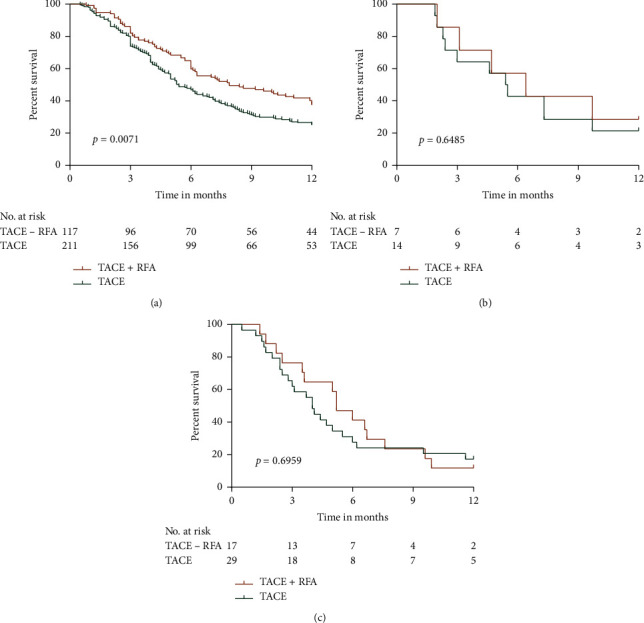
Comparisons of the survival of TACE + RFA and TACE alone. (a) PVTT; (b) HVTT; and (c) PVTT + HVTT.

The 12-month OS rates of HCC patients with PVTT, HVTT, or PVTT + HVTT were also analyzed. Our results demonstrated that TACE + RFA treatment contributed to a significantly higher rate of 12-month survival in HCC patients with PVTT compared to those with TACE treatment (*p*=0.0071, Figure 6(a)). However, the contributions of TACE + RFA and TACE treatments to 12-month survival did not differ considerably in HCC patients with HVTT (Figure 6(b)) and PVTT + HVTT (Figure 6(c)) (*p*=0.6485 and *p*=0.6959, respectively).

## 4. Discussion

Intermediate stage HCC is commonly treated with a combination of TACE and RFA [18, 19]; however, the effectiveness of this treatment, compared to that of TACE treatment alone, in the advanced stage HCC patients is still unknown. The present study compared the effectiveness of TACE + RFA treatment and TACE treatment in HCC patients with MVI. Our results indicated that TACE + RFA prolonged OS in HCC patients with MVI. MVI in the portal and hepatic veins highly correlates with the degree of tumor malignancy [20, 21]. The study demonstrated that AFP <400 ng/ml, tumor number <3, tumor diameter <5 cm, or PVTT patients who received TACE + RFA treatment showed considerably higher 12-month OS rates than those who received TACE treatment (*p*=0.0122, *p*=0.0090, *p*=0.0112, and *p*=0.0071, respectively); however, both treatments contributed to similar OS rates in AFP ≥ 400 ng/ml, tumor number ≥ 3, tumor diameter ≥ 5 cm, HVTT or PVTT + HVTT patients (*p*=0.4208, *p*=0.6478, *p*=0.4700, *p*=0.6485, and *p*=0.6959, respectively).

Nowadays, effective treatments for HCC patients with MVI are limited and controversial. International guidelines recommend sorafenib as the only treatment for HCC patients with MVI [22, 23]. Nevertheless, patients treated with sorafenib show a median OS of 8.1 months and tend to have a low tolerance to the drug [9]. Therefore, its curative effects have been questioned in HCC patients with MVI without extrahepatic proliferation. However, surgery, as the first choice, can be used to treat HCC in the early phase while for HCC, which involves the main portal vein or the main branches, surgery is not proper. According to many studies, RFA and TACE are likely to be beneficial for the unresectable HCC regarding local control.

TACE is the preferred choice in patients with unresectable HCC. The expert consensus statement of the 2010 International Hepato-Pancreato-Biliary Association defined TACE as a standard therapeutic approach for unresectable HCC, regardless of portal vein involvement (main portal vein excluded) [24]. TACE has been reported to show better survival rates than conservative therapy in HCC patients with PVTT [25–27]. In the present study, unresectable HCC included middle and advanced tumor with poor liver function reserve, more than 3 tumor nodules that were localized to the different segment or lobe, and extrahepatic metastases. According to our retrospective study, TACE showed better survival benefits in HCC patients with PVTT than conservative treatment. Despite delayed tumor progression and enhanced OS due to ischemic necrosis caused by arterial embolization, TACE could hardly achieve complete necrosis in the target lesion. Following TACE, incomplete embolization may result in intrahepatic or extracapsular tumor invasion. Despite the safety and effectiveness of TACE in chosen HCC patients with MVI, median survival time remains 3.8–9.5 months [25]. In addition, it is reported that 27–63.2% of patients in advanced HCC stage saw an AP shunt [28, 29], due to PVTT [30]. AP shunts sharpen the complications presented by portal hypertension, such as refractory ascites and esophageal varices [23, 31]. Polyvinyl alcohol [32], N-butyl cyanoacrylate [33], and ethanol-soaked gelatin sponge [34] have been used for the treatment of AP shunts. In this study, gelatin sponge particles were used to treat AP shunts.

RFA is highly suitable for treating unresectable HCC and can achieve better results, especially in HCCs with diameters <4 cm [29]. However, owing to failure in achieving complete necrosis or optimal local tumor control, it is not suitable for treating HCCs with diameters >5 cm [35]. Nevertheless, it has been reported in recent years that RFA can be used to treat HCC with PVTT [36].

Compared to single treatment with either RFA or TACE, combining TACE with RFA provides several advantages. First, TACE can improve the ablation rates of bigger tumors by reducing the tumor burden and reducing the viable tumor volume prior to RFA. Moreover, TACE or repeated TACE may narrow and even occlude the major supplying arteries to the tumor [37], adding to the difficulty of selective catheterization of the feeding artery for controlling the residual tumor cells. Furthermore, HCC is a tumor rich in blood supply. RFA was easily affected by blood vessel-mediated cooling (the heat-sink effect) [38], a significant influencing factor for hepatic malignant tumor recurrence following RFA [39]. TACE can reduce or block the hepatic artery blood flow, thus reducing the heat loss during RFA and may increase the volume of the zone of ablation and complete ablation rate [35, 40, 41]. Finally, subsequent RFA would contribute to a direct ablation effect on the refractory tumors. A combination of TACE and RFA is effective for local control of medium-sized HCCs (3–5 cm) and HCC patients with PVTT [41, 42]. Nevertheless, the effectiveness of this combined therapy remains unknown in HCC patients with MVI. Therefore, this study assessed the efficacy and survival rates related to TACE + RFA method for treating advanced HCC.

There were some limitations to this study. First, this study was conducted retrospectively and did not involve any randomization of the study participants. Second, all TACE and RFA procedures were performed in a single institution. Therefore, the experiences of patients and physicians could affect the study results. Third, different from those conducted in the United States, Japan, and Europe, the study found that 73% of the patients had hepatitis B virus infection. Hence, it is necessary to further investigate therapeutic strategies in HCC patients in the abovementioned areas. Fourth, no standardized treatment schemes of TACE are available regarding anticancer agent dosage, treatment type, and schedule. Applying a nonstandardized treatment scheme restricts therapeutic efficacy prediction. Fifth, the better OS for TACE and RFA treatment might be simply due to the fact that cases subjected to RFA could have tumors located in better position, not so close to a major portal vein, where RF would have been contraindicated; this could be demonstrated by the fact that no differences have been shown in cases with AFP greater than 400 ng/ml where it is highly probable that major hepatic vessels were in close proximity to the tumors not allowing RFA. This possibility might have produced a bias selection.

## 5. Conclusions

In summary, the results of our study showed that compared to TACE, TACE + RFA could be more effective for treating HCC patients with MVI, especially AFP <400 ng/ml, tumor number <3, tumor diameter <5 cm, or PVTT patients because it could hinder tumor progression and increase OS. However, TACE + RFA and TACE alone showed similar effects in patients with AFP ≥400 ng/ml, tumor number ≥3, tumor diameter ≥5 cm, HVTT, or PVTT + HVTT.

## Figures and Tables

**Table 1:** Baseline characteristics of patients before matching.

Variables	Control	TACE	*p* value	TACE + RFA	*p* value
	(*n* = 269)	(*n* = 254)		(*n* = 141)	
Median age (range)	55 (25–81)	55 (25–78)	0.695^a)^	56 (28–78)	0.241^a)^
Sex (M/F)	231/38	217/37	0.886^b)^	117/24	0.437^b)^
Family history of HCC (yes/no)	38/231	30/224	0.431^b)^	15/126	0.317^b)^
HBV related (yes/no)	248/21	233/21	0.846^b)^	129/12	0.803^b)^
GGT (IU/L)	185.3 (90.7–322.2)	125.5 (80.3–181.1)	<0.001^c)^	95.2 (59.5–143.4)	<0.001^c)^
WBC (×10^9^/L)	5.0 (3.6–6.6)	4.6 (3.3–5.9)	0.027^c)^	4.5 (3.5–6.5)	0.559^c)^
PLT (×10^9^/L)	108.4 (69.9–158.8)	93.1 (62.0–144.3)	0.068^c)^	112.9 (64.0–161.6)	0.747^c)^
AFP (ng/mL) (≥400/<400)	138/131	114/140	0.142^b)^	55/86	0.018^b)^
PVTT (%)	226 (84.0)	211 (83.1)	0.849^b)^	117 (83.0)	0.849^b)^
HVTT (%)	12 (4.5)	14 (5.5)	0.757^b)^	7 (5.0)	0.987^b)^
PVTT + HVTT (%)	31 (11.5)	29 (11.4)	0.844^b)^	17 (12.1)	0.979^b)^
Tumor number (≥3/<3)	160/109	111/143	<0.001^b)^	48/93	<0.001^b)^
Tumor diameter (cm) (≥5/<5)	167/102	154/100	0.733^b)^	77/64	0.143^b)^
Child-Pugh class (A/B or C)	69/200	133/121	<0.001^b)^	89/52	<0.001^b)^
MELD score	9.1 (5.3–11.9)	6.4 (3.9–8.4)	<0.001^c)^	6.0 (3.0–8.3)	<0.001^c)^
TNM (III/IV)	221/48	211/43	0.783^b)^	125/16	0.085^b)^
BCLC (C/D)	202/67	225/29	<0.001^b)^	129/12	<0.001^b)^

Age is presented as median (range), categorical variables as number (%), continuous variable as mean (interquartile range). a), *t*-test; b), chi-square test or Fisher's exact test; c), Mann–Whitney *U* test. HBV, hepatitis B virus; GGT, gamma-glutamyl transferase; WBC, white blood cell count; PLT, platelet count; AFP, *α*-fetoprotein; PVTT, portal vein tumor thrombosis; HVTT, hepatic vein tumor thrombosis; MELD, model for end-stage liver disease; TNM, tumor, node, and metastasis staging; BCLC, Barcelona Clinic for Liver Cancer.

**Table 2:** Baseline characteristics of patients after matching.

Variables	TACE	Control	*p* value	TACE + RFA	Control	*p* value
	(*n* = 188)	(*n* = 188)		(*n* = 102)	(*n* = 102)	
Median age (range)	55 (25–78)	54 (25–81)	0.655^a)^	57 (28–78)	53 (25–80)	0.068^a)^
Sex (M/F)	164/24	160/28	0.500^b)^	84/18	90/12	0.236^b)^
Family history of HCC (yes/no)	24/164	25/163	0.878^b)^	10/92	15/87	0.286^b)^
HBV related (yes/no)	169/19	174/14	0.362^b)^	93/9	94/8	0.800^b)^
GGT (IU/L)	169.4 (86.8–190.4)	197.9 (83.2–275.3)	0.141^c)^	101.5 (59.7–156.4)	112.2 (60.4–185.3)	0.711^c)^
WBC (×10^9^/L)	5.0 (3.4–5.8)	5.1 (3.5–6.4)	0.319^c)^	4.5 (3.6–6.7)	5.2 (3.8–6.3)	0.955^c)^
PLT (×10^9^/L)	112.6 (61.7–143.8)	126.9 (70.3–162.3)	0.027^c)^	111.8 (63.1–161.5)	108.7 (69.9–164.4)	0.898^c)^
AFP (ng/mL) (≥400/<400)	90/98	88/100	0.836^b)^	44/58	41/61	0.670^b)^
PVTT (%)	154 (81.9)	147 (78.2)	0.480^b)^	83 (81.4)	85 (83.3)	0.713^b)^
HVTT (%)	11 (5.9)	12 (6.4)	1.000^b)^	6 (5.9)	6 (5.9)	1.000^b)^
PVTT + HVTT (%)	23 (12.2)	29 (15.4)	0.535^b)^	13 (12.7)	11 (10.8)	0.663^b)^
Tumor number (≥3/<3)	92/96	103/85	0.256^b)^	41/61	38/64	0.666^b)^
Tumor diameter (cm) (≥5/<5)	109/79	122/66	0.167^b)^	56/46	67/35	0.115^b)^
Child-Pugh class (A/B or C)	79/109	66/122	0.168^b)^	53/49	50/52	0.674^b)^
MELD score	6.8 (4.4–8.8)	7.7 (4.8–9.6)	0.672^c)^	6.4 (3.4–8.8)	7.4 (4.6–9.3)	0.079^c)^
TNM (III/IV)	153/35	155/33	0.789^b)^	88/14	88/14	1.000^b)^
BCLC (C/D)	160/28	162/26	0.769^b)^	92/10	87/15	0.286^b)^

Age is presented as median (range), categorical variables as number (%), continuous variable as mean (interquartile range). a), *t*-test; b), chi-square test or Fisher's exact test; c), Mann–Whitney *U* test. HBV, hepatitis B virus; GGT, gamma-glutamyl transferase; WBC, white blood cell count; PLT, platelet count; AFP, *α*-fetoprotein; PVTT, portal vein tumor thrombosis; HVTT, hepatic vein tumor thrombosis; MELD, model for end-stage liver disease; TNM, tumor, node, and metastasis staging; BCLC, Barcelona Clinic for Liver Cancer.

**Table 3:** Baseline characteristics of patients undergoing TACE or TACE + RFA before and after matching.

Variables	Before matching			After matching		
	TACE	TACE + RFA	*p* value	TACE	TACE + RFA	*p* value
	(*n* = 254)	(*n* = 141)		(*n* = 139)	(*n* = 139)	
Median age (range)	55 (25–78)	56 (28–78)	0.131^a)^	54 (25–78)	56 (28–78)	0.183^a)^
Sex (M/F)	217/37	117/24	0.518^b)^	112/27	117/22	0.431^b)^
Family history of HCC (yes/no)	30/224	15/126	0.725^b)^	15/124	14/125	0.844^b)^
HBV related	232/22	129/12	0.959^b)^	129/10	127/12	0.657^b)^
WBC (×10^9^/L)	4.9 (3.3–5.8)	4.5 (3.5–6.5)	0.234^c)^	4.7 (3.5–6.0)	4.6 (3.6–6.6)	0.542^c)^
PLT (×10^9^/L)	114.7 (62.0–144.5)	112.9 (64.0–161.6)	0.287^c)^	98.0 (66.0–143.3)	113.9 (65.0–161.8)	0.542^c)^
AFP (ng/mL) (≥400/<400)	114/140	55/86	0.258^b)^	54/85	54/85	1.000^b)^
PVTT (%)	211 (83.1)	117 (83.0)	1.000^b)^	119 (85.6)	119 (85.6)	1.000^b)^
Vp1	3 (1.2)	2 (1.4)	1.000^b)^	1 (0.7)	2 (1.4)	0.562^b)^
Vp2	17 (6.7)	15 (10.6)	0.323^b)^	14 (10.1)	14 (10.1)	1.000^b)^
Vp3	66 (26.0)	44 (31.2)	0.434^b)^	53 (38.1)	51 (36.7)	0.804^b)^
Vp4	125 (49.2)	56 (39.7)	0.200^b)^	64 (46.0)	65 (46.8)	0.904^b)^
HVTT (%)	14 (5.5)	7 (5.0)	0.769^b)^	7 (5.0)	6 (4.3)	0.776^b)^
PVTT + HVTT (%)	29 (11.4)	17 (12.1)	0.825^b)^	13 (9.4)	13 (9.4)	1.000^b)^
Tumor number (≥3/<3)	111/143	48/93	0.061^b)^	47/92	47/92	1.000^b)^
Tumor diameter (cm) (≥5/<5)	156/98	77/64	0.188^b)^	88/51	76/63	0.143^b)^
Child-Pugh class (A/B or C)	136/118	89/52	0.065^b)^	89/50	89/50	1.000^b)^
MELD score	6.4 (3.9–8.4)	6.0 (3.0–8.3)	0.159^c)^	5.6 (3.7–7.8)	5.1 (3.1–8.3)	0.812^c)^
TNM (III/IV)	211/43	125/16	0.136^b)^	114/25	123/16	0.128^b)^
BCLC (C/D)	225/29	129/12	0.364^b)^	126/13	128/11	0.669^b)^

Age is presented as median (range), categorical variables as number (%), continuous variable as median (interquartile range). a), *t*-test; b), chi-square test or Fisher's exact test; c), Mann–Whitney *U* test. HBV, hepatitis B virus; GGT, gamma-glutamyl transferase; WBC, white blood cell count; PLT, platelet count; AFP, *α*-fetoprotein; PVTT, portal vein tumor thrombosis; HVTT, hepatic vein tumor thrombosis; MELD, model for end-stage liver disease; TNM, tumor, node, and metastasis staging; BCLC, Barcelona Clinic for Liver Cancer.

## Data Availability

The datasets generated and analyzed during the current study are available from the corresponding author upon reasonable request.
